# *Helicobacter pylori* Breath Test via Mid-Infrared Sensor Technology

**DOI:** 10.1021/acssensors.4c02785

**Published:** 2025-02-08

**Authors:** Gabriela Flores Rangel, Lorena Diaz de León Martinez, Boris Mizaikoff

**Affiliations:** † Institute of Analytical and Bioanalytical Chemistry, Ulm University, Albert-Einstein-Allee 11, Ulm 89081, Germany; ‡ Hahn-Schickard, Sedanstrasse 14, Ulm 89077, Germany

**Keywords:** Helicobacter pylori, exhaled breath testing, IR spectroscopy, substrate-integrated hollow waveguide, iHWG, mid-infrared, MIR, noninvasive diagnostic, ^12^CO_2_, ^13^CO_2_, breath biomarkers, quantum cascade laser, QCL

## Abstract

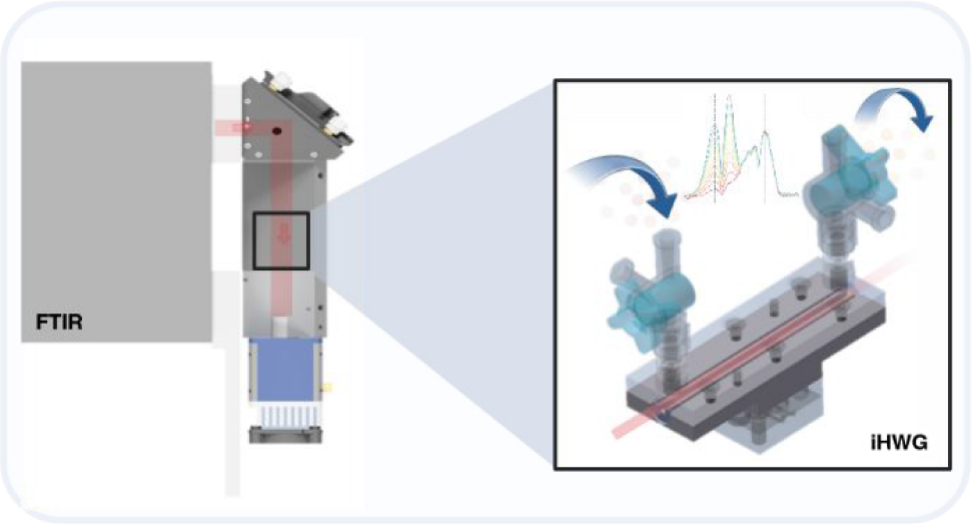

*Helicobacter pylori* infection has been associated with various gastrointestinal disorders, most notably with the development of gastric cancer. Therefore, it is important to develop technologies for effective, rapid, sensitive, and personalized infection detection. The present study evaluates the utility of mid-infrared (MIR) exhaled breath sensors utilizing substrate-integrated hollow waveguide (iHWG) technology for the precise determination of the isotopic ratio of ^13^CO_2_ vs ^12^CO_2_ simulating conditions relevant to the detection of the presence of *Helicobacter pylori* in the upper gastrointestinal tract via exhaled breath analysis. For future integration of such a sensing module, e.g., into a cell phone attachment, optimized light–gas interaction and sufficient sensitivity are essential, as the diagnosis is based on detecting the presence of ^13^CO_2_ 30 min after administration of ^13^C-labeled urea via a gel or pill, which is metabolized by *H. pylori*. By optimizing the light–gas interaction volume via tailoring of the iHWG, it was demonstrated that sufficient sensitivity and accuracy are achieved for detecting small changes in the isotopic composition of exhaled CO_2_. While it was demonstrated that the combination of conventional Fourier-transform infrared (FTIR) spectroscopy with iHWGs indeed confirms the utility of this noninvasive breath analysis concept, further device miniaturization utilizing quantum cascade lasers is anticipated to achieve the necessary level of integration for personalized home usage.

*Helicobacter pylori* (*H. pylori*) infection has been extensively associated with gastrointestinal diseases, most notably gastritis, peptic ulcers, and gastric cancer. Consequently, an accurate diagnosis of *H. pylori* infection is critical to prevent disease progression and guide treatment, particularly in patients with a history of ulcer disease where direct eradication therapy^1−3^ is essential. A variety of diagnostic methods are available, each with specific benefits and limitations. Invasive methods, such as endoscopic biopsy followed by histological examination or bacterial culture, offer high accuracy but require patient sedation and specialized equipment, making these strategies both costly and uncomfortable.^4^

Current diagnostic methods for *H. pylori* infection^5^ emphasize the drawbacks of invasive techniques and the pressing need for accurate, noninvasive alternatives. Among these alternatives, paper-based microfluidic biosensors have gained significant attention due to their cost-effectiveness, simplicity, and portability, making them particularly attractive for point-of-care applications. These systems^6^ leverage electrochemical detection mechanisms within paper substrates, providing a lightweight and disposable format suitable for resource-limited settings. While this approach offers notable advantages, challenges remain in achieving the high sensitivity and precision required for applications involving isotopic gas sensing.

Other noninvasive diagnostic options for *H. pylori* include stool antigen tests, serology tests, rapid urease tests, histology, bacterial culture, molecular tests, and breath tests.^7−9^ Among these, the ^13^C-urea breath test (UBT) stands out as a highly innovative strategy due to its accuracy and noninvasiveness. This test exploits the bacterial ability to hydrolyze urea via the enzyme urease, producing labeled carbon dioxide (^13^CO_2_) when ^13^C-labeled urea is ingested. The presence of ^13^CO_2_ results from this enzymatic conversion, which occurs only if an *H. pylori* infection is present. By analyzing the ratio of ^13^CO_2_ to ^12^CO_2_ in the exhaled breath matrix, it can be determined whether *H. pylori* is active.^10,11^

Carbon dioxide (CO_2_) plays a fundamental role in breath analysis as it is abundantly present in exhaled air, primarily produced by the body’s metabolic processes. Isotopic breath tests, such as the ^13^C-urea breath test, utilize ^13^CO_2_, a naturally stable isotope of carbon, as a noninvasive biomarker.^12−14^ While ^12^CO_2_ accounts for over 98% of all naturally occurring CO_2_, the lower abundance of ^13^CO_2_ (∼1%) makes isotopic ratio analysis a sensitive method for detecting subtle biological changes.^13^ In this context, both ^13^CO_2_ and ^12^CO_2_ serve as key analytes in evaluating *H. pylori* metabolic activity via breath analysis.

Isotopic ratio of ^13^CO_2_ vs ^12^CO_2_ is frequently expressed as a “delta value”, which represents the deviation of the isotopic ratio of the sample compared to a standard reference material with a constant natural ^13^C content, i.e., typically, Vienna Pee Dee Belemnite (VPDB). This delta value is calculated using^14^
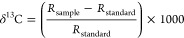
where *R*_sample_ is the ratio of ^13^CO_2_ vs ^12^CO_2_ in the sample, and *R*_standard_ is the isotopic ratio in the VPDB standard.

The delta value provides a normalized way to express the isotopic composition of the sample, allowing for the comparison across different measurements and experimental conditions. In the context of *H. pylori* detection, a significant increase in δ^13^C after ingestion of ^13^C-labeled urea indicates the presence of an active infection, as the bacterial urease breaks down the urea, leading to an elevated production of ^13^CO_2_. By determining changes in δ^13^C, it is even possible to quantify the metabolic activity of *H. pylori*.^15^ Currently, the equipment needed for analyzing breath samples, particularly for isotopes such as ^13^C, is based on mass spectrometric or infrared spectroscopic techniques, which can be expensive, require trained personnel for operation, and may not be widely available, limiting accessibility in some healthcare settings.

However, the clinical implementation of breath tests requires addressing factors that may affect measurement accuracy, such as other urease-producing bacteria, variations in gastric emptying rates, the use of proton pump inhibitors or antibiotics, and changes in gastric pH. Strategies to mitigate these issues include implementing a fasting period before the test, using citric acid with ^13^C-urea, restricting certain medications, collecting multiple breath samples, establishing cutoff values, and employing composite reference methods.^16,17^

Despite the proven efficacy of mass spectrometric and infrared spectroscopic techniques for analyzing isotopic ratios, their high cost, operational complexity, and limited accessibility in some healthcare settings pose challenges. Recent advancements in biosensing technologies have focused on miniaturization, portability, and point-of-care diagnostics to overcome these barriers. For instance, bioelectrochemical principles have enabled the creation of biosensors with enhanced sensitivity and specificity.^18^ Similarly, advances in multiplex biosensing^19^ devices have paved the way for comprehensive and rapid diagnostics, aligning with the design principles of substrate-integrated hollow waveguides (iHWGs).

Mid-infrared (MIR) spectroscopy is an established technique for detecting and quantifying gas-phase analytes and is particularly suitable for analyzing strongly IR-absorbing molecules such as CO_2_, which have distinct absorption bands in the MIR range.^20^ The isotopologues ^13^CO_2_ and ^12^CO_2_ exhibit sufficiently discriminatory absorption peaks due to their mass difference, with ^12^CO_2_ absorbing at ∼2349 cm^–1^ and ^13^CO_2_ absorbing at a lower frequency (∼2280 cm^–1^). This difference allows for the precise discrimination and quantification of the two isotopes rendering MIR spectroscopy an ideal tool for isotopic ratio analysis.^21^ In contrast to, e.g., mass spectrometry, IR techniques are particularly amenable to miniaturization facilitating the development of highly portable, sensitive, and specific sensing devices that provide a signal response within minutes offering a viable alternative to address accessibility needs, especially in low-resource healthcare settings.^22^

The aim of the present study was to evaluate the utility of Fourier-transform infrared (FTIR) spectroscopy to accurately determine the ^13^CO_2_/^12^CO_2_ ratio in combination with innovative substrate-integrated hollow waveguides (iHWGs)^20,22−24^ enhancing the light–gas interaction. This technology allows later replacement of the IR spectrometer with quantum cascade lasers (QCLs), interband cascade lasers (ICLs), or even interband cascade LEDs (ICLEDs) emitting at selected wavelengths in the MIR for establishing highly miniaturized *H. pylori* breath test modules, potentially in the format of a simple cell phone attachment. By optimizing the interaction volume of the iHWG by tailoring its length, it was demonstrated that the iHWG—as the key component where the analytical signal is generated—allows optimizing the detection capabilities toward a noninvasive diagnostic tool for ^13^C-urea breath testing^25^ in a possibly compact format.

## Materials and Methods

The iHWG assembly consists of two aluminum components: the base plate with the machined channel and a top plate that acts as a lid. These parts are bonded together using an epoxy adhesive, ensuring a permanent and hermetic seal. The ends of the waveguides are hermetically sealed with mid-infrared-transparent BaF_2_ windows, creating a miniaturized gas cell. Threaded ports for gas inlets and outlets are incorporated into the top plate to facilitate the efficient flow of the sample gas through the radiation propagation channel.

### Experimental Conditions

No patient samples were analyzed in this study. Instead, synthetic gas mixtures of ^12^CO_2_ and ^13^CO_2_ were prepared in controlled concentrations to simulate isotopic breath test conditions. This approach ensured reproducibility and allowed for precise evaluation of the iHWG-based detection system’s performance under laboratory conditions.

A compact FTIR spectrometer (Alpha II, Bruker Optik GmbH, Ettlingen, Germany) was used for the experiments. The FTIR system was operated at a spectral resolution of 2 cm^–1^ recording spectra across a spectral range of 1800–2600 cm^–1^. Each measurement averaged 64 spectral scans with a total signal acquisition time of 140 s. All measurements were collected with 7 replicas. A synthetic air background measurement was collected prior to each experiment; between each sample measurement, the system was flushed with synthetic air for 2 min.

For gas handling, a set of mass flow controllers (Bronkhorst, AK Ruurlo, Netherlands) was used to maintain a constant total gas flow of 500 mL/min. The gas mixtures consisted of ^12^CO_2_ (1%) in synthetic air and ^13^CO_2_ (1%) in synthetic air. These controlled gas mixtures allowed for the simulation of conditions relevant to isotopic breath analysis. The mixtures were prepared in a range from 10 to 1000 ppm for ^13^CO_2_ and from 100 to 1000 ppm for ^12^CO_2_.

### Instrumentation and Setup

**Figure 1:**
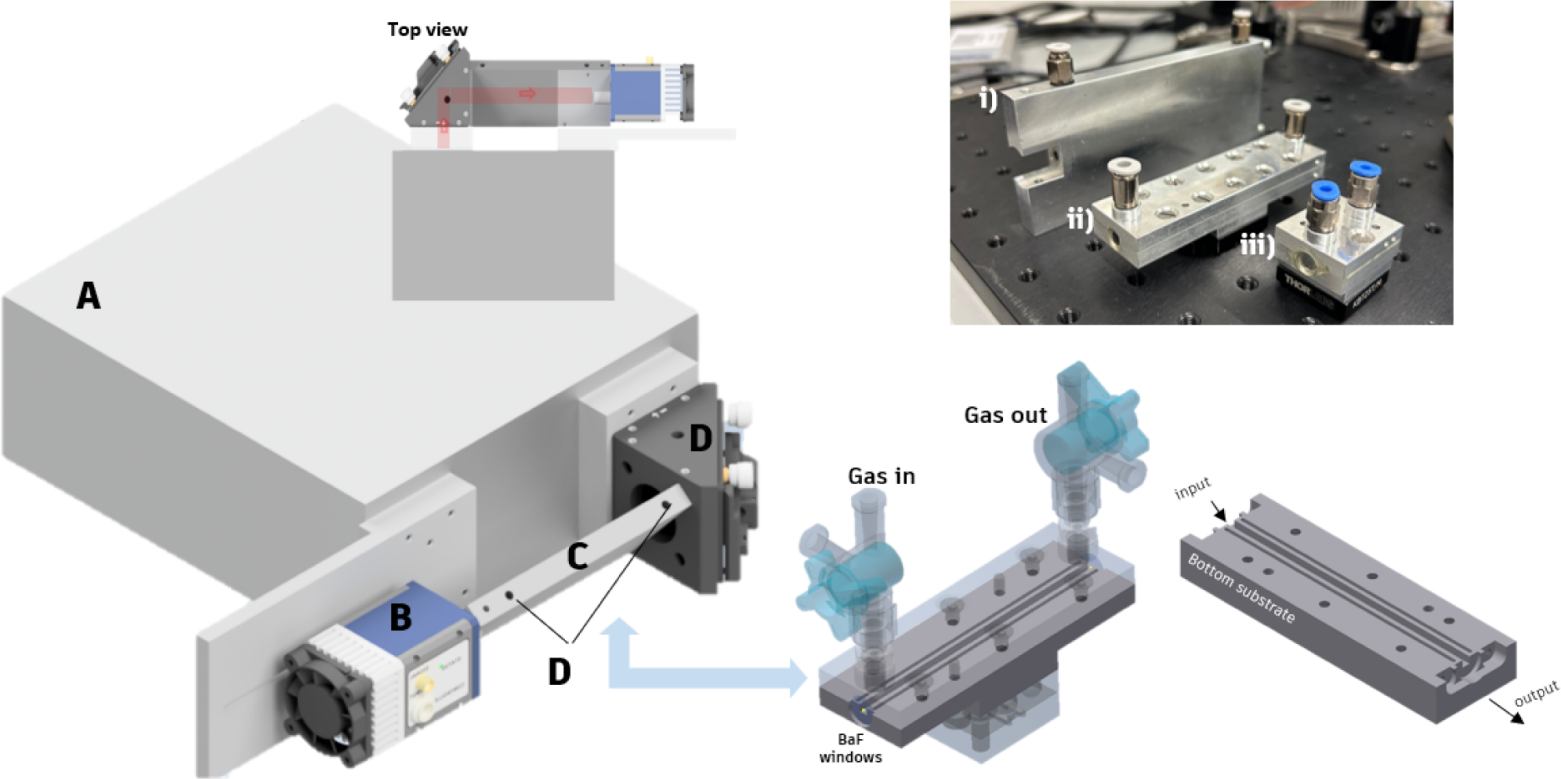
Left: (A) FTIR spectrometer, (B) MCT detector, (C) iHWG, (D) gas inlet/outlet, mirror holder with integrated off-axis parabolic mirror. Upper right: integrated hollow waveguides (iHWGs) with lengths of (i) 10.5 cm, (ii) 9 cm, and (iii) 3 cm. Lower right: CAD iHWG design.

The FTIR spectrometer was coupled with a custom-designed iHWG, as previously reported.^24^ The entire sensing setup, including the iHWG and FTIR spectrometer, occupies a footprint of approximately 30 cm × 20 cm × 15 cm, supporting the feasibility of developing portable diagnostic devices. Three different wavelengths were investigated: 3 cm (rectangular core), 9 cm (rectangular core), and 10.5 cm (circular core) (Figure 1, upper right). The corresponding internal volumes for each iHWG were calculated as follows:

- A—3 cm length: 480 μL
- B—10.5 cm length: 1.31 mL
- C—9 cm length: 1.44 mL

These waveguides served simultaneously as highly efficient gas cells, enhancing the interaction between the MIR radiation and the gaseous molecules, with longer waveguides increasing the interaction volume for optimizing the detection of small changes in ^13^CO_2_/^12^CO_2_ ratio. In turn, reduced iHWG lengths facilitate faster sample exchange/response times and also allow a possibly flexible optimization of the system performance adapted to the application scenario

### Detection System

For MIR radiation detection, an external mercury cadmium telluride (MCT) detector (Vigo Infrared-Detector, model PVI-4TE-8-1x1) was used. The sensing system along with the optical arrangement is shown in Figure 1 (left). Throughout the experiments, the flow velocity of the gas mixtures was set at a constant flow rate of 500 mL/min controlled via a set of mass flow controllers. After each measurement, the system was flushed with synthetic air (500 mL/min) for 1–2 min, ensuring the removal of residual gas from the previous measurement.

### Calibration and Data Analysis

For both gases, calibration functions were established for determining the analytical figures of merit, including the limit of detection (LOD), the limit of quantification (LOQ), the sensitivity (slope *m* of the calibration function), and the linearity. The MIR data acquisition parameters were based on previous studies.^26^ For data acquisition, the OPUS 8.5 software package (Bruker Optik GmbH, Ettlingen, Germany) was used; data processing was performed using the ORIGIN PRO software package.

## Results and Discussion

The obtained analytical figures of merit for each analyte using the different iHWGs are summarized in Table 1. For ^12^CO_2_, the peak at 2360 cm^–1^, and for ^13^CO_2_ the peak at 2270 cm^–1^ were selected for peak height analysis. It should be noted that the benchmark value to be detected is that the ratio ^13^CO_2_/^12^CO_2_ exceeds 4‰, which means that a patient is considered positive with typical concentrations of around 16 ppm of ^13^CO_2_ and 884 ppm of ^12^CO_2_; this is an example of a positive infection indication.^27^

**Table 1:** Analytical Figures of Merit for ^12^CO_2_ and ^13^CO_2_ Using Three Different iHWGs

Gas	iHWG	LOD (ppm)	LOQ (ppm)
^13^CO_2_ 10–100 ppm	A	4.84	16.15
	B	1.44	4.82
	C	6.36	21.21
^13^CO_2_ 100–1000 ppm	A	37.85	126.16
	B	90.1	300.33
	C	79.86	266.21
^12^CO_2_ 100–1000 ppm	A	58.87	196.26
	B	34.48	114.96
	C	27.52	91.73

Considering the benchmark values, each iHWG exhibits suitable detection limits for the application scenario discussed herein. All investigated iHWGs operate effectively even at the lowest concentrations (i.e., <10 ppm). Hence, even for the 3-cm iHWG, the LODs are lower than necessary for diagnosing a weakly positive infection (4.84 ppm for ^13^CO_2_ and 58.84 ppm for ^12^CO_2_), which indicates that a highly miniaturized sensor using a QCL, ICL, or ICLED can indeed be envisaged. Exemplary IR spectra from each iHWG and the individual gases are given in the Supporting Information, where it is important to note that for the same set of concentrations, all iHWGs, even the lowest volume device at 480 μL, exhibit suitable absorption signals allowing for the efficient detection of the evaluated compounds.^28−30^

**Figure 2:**
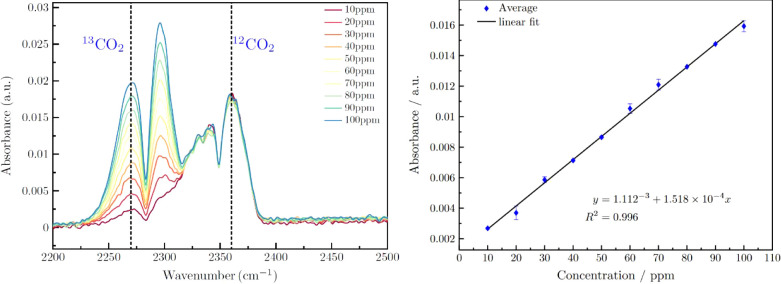
Left: Exemplary IR spectra show the ^13^CO_2_/^12^CO_2_ ratios in 10-cm iHWGs, with ^12^CO_2_ fixed at 200 ppm and varying ^13^CO_2_ concentrations from 10 to 100 ppm. Right: Calibration curve was derived from the mixture as a function of ^13^CO_2_ concentration.

To further corroborate these findings and evaluate the ^13^CO_2_/^12^CO_2_ ratio as closely as possible to the values reported for individuals with *H. pylori*, further calibrations were established by varying the concentration of ^13^CO_2_ against fixed concentrations of 200 and 100 ppm of ^12^CO_2_. The peak observed around 2300 cm^–1^ in Figure 2 corresponds to the asymmetric stretching vibration mode (ν_3_) of carbon dioxide in its most abundant isotopic form, ^12^CO_2_. Although increasing the concentration of ^13^CO_2_ may cause slight variations in the intensity of this peak due to spectral overlap effects, our primary focus is on the ^13^CO_2_ peak, located at approximately 2280 cm^–1^, which serves as the relevant isotopic marker for detecting urease activity associated with *Helicobacter pylori*. This overlapping phenomenon between the isotopologues reflects the proximity of their absorption frequencies due to the isotopic shift and underscores the importance of employing multivariate analysis models to separate individual contributions and ensure reliable measurements. Figure 2 (left panel) illustrates how the peaks corresponding to both isotopologues vary with changing ^13^CO_2_ concentrations, while the right panel presents a linear calibration curve based on the ^13^CO_2_ peak, confirming the system’s ability to discriminate and quantify this isotopologue in simulated breath gas mixtures. While a threshold evaluation for an *H. pylori* infection is certainly possible, it is evident that the ^12^CO_2_ signature is at least slightly affected by the spectrum of ^13^CO_2_ at increasing concentrations of the latter. Hence, for a reliable and precise quantification in mixtures, more sophisticated data evaluation models have to be established taking spectral overlap and matrix effects into account. The substitution of ^12^C by ^13^C in the CO_2_ molecule causes a shift of the absorption frequency due to the mass difference; however, the magnitude of the shift is not sufficient to prevent overlap of the corresponding bands. Consequently, as the concentration of ^13^CO_2_ increases while maintaining a constant ^12^CO_2_ concentration, a slight increase in the intensity of the ^12^CO_2_ spectrum is observed. This behavior is particularly relevant for the precise quantification of ^13^CO_2_ within breath tests, which is less crucial for a rather simple yes/no decision. However, a more sophisticated multivariate calibration model is currently in development facilitating a more precise quantification in application scenarios where this is indeed needed.

**Figure 3:**
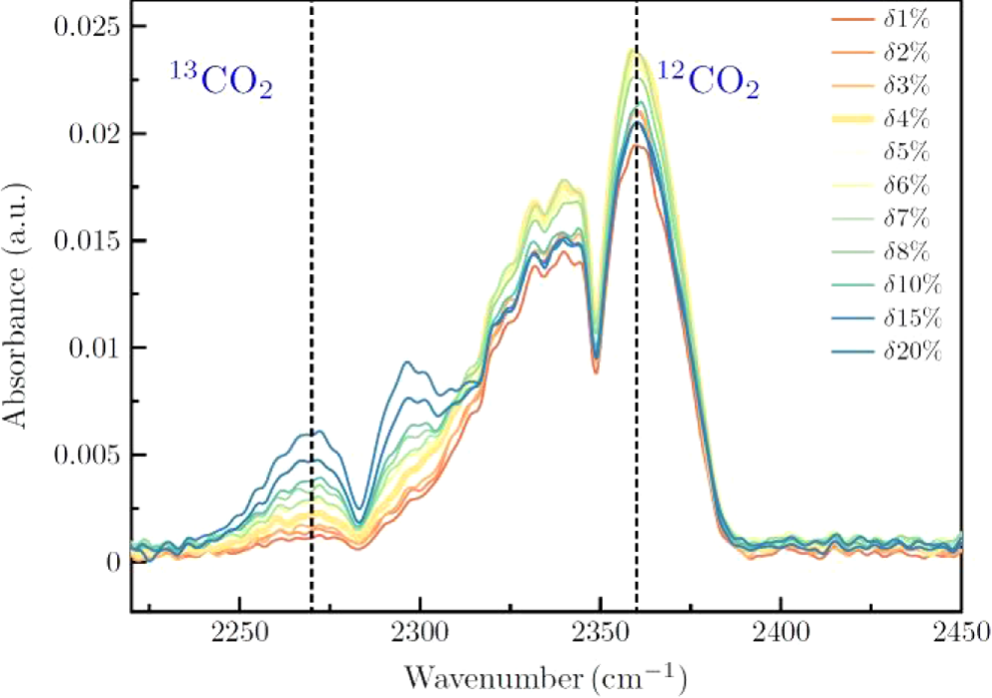
Absorbance spectra of ^13^CO_2_ and ^12^CO_2_ at various δ values (1–20%). The graph illustrates how the δ^13^C (‰) ratio varies across different concentrations, with the threshold for detection at δ = 4% indicated as a reference. These measurements were obtained using 480 μL iHWG.

In order to demonstrate the effectiveness of the ^13^CO_2_/^12^CO_2_ ratio analysis for future exhaled breath studies and to validate the use of 480 μL of iHWG, the corresponding δ^13^C (‰) values were calculated for different gas ratios. The results, displayed in Figure 3, show the outcomes at various concentrations, correlating with different δ values. The threshold for *Helicobacter pylori* detection, indicated by the green line at δ = 4‰, differentiates between the absence and presence of the bacteria. These results highlight the utility of iHWG-based sensors in achieving sufficient sensitivity with relatively small sample volumes. In comparison to other studies, which have reported the use of sample volumes as small as 250 μL, 1.5 mL, and 200 mL for breath analysis using IR spectroscopy, our results confirm that adequate sensitivity can be maintained even with a reduced volume. By employing miniaturized IR light sources, this approach paves the way for portable device platforms, facilitating early infection detection.

## Conclusions

This study demonstrated the utility of substrate-integrated hollow waveguide-based IR-spectroscopic sensing concepts for the detection of ^12^CO_2_ and ^13^CO_2_, with particular emphasis on minimizing the required gas sample volume in personalized medical devices. It was shown that even the smallest iHWG volume allows for exceedingly low detection limits <10 ppm for the target species ^13^CO_2_. Hence, an iHWG with a gas sensing volume of 480 μL still provides sufficient light–gas interaction, paving the way toward compact and portable *H. pylori* infection detection systems.

The clinically relevant δ^13^C (‰) value confirmed that the 3-cm-long iHWG may accurately distinguish between positive and negative cases for *H. pylori* diagnosis, beyond the detection limits required for diagnosing positive cases (4.84 ppm for ^13^CO_2_ and 58.84 ppm for ^12^CO_2_). This renders MIR gas sensor systems ideally suited for noninvasive on-site diagnostic applications, particularly for breath tests aiming at detecting *H. pylori* infections.

However, the observed spectral overlap and matrix effects when analyzing mixtures of ^12^CO_2_ and ^13^CO_2_ complicate precise quantification and demand more sophisticated multivariate data evaluation strategies taking these aspects into account. A main aspect of the present study was to substantiate that the spectral differences between ^12^CO_2_ and ^13^CO_2_ are sufficient for their discrimination after the administration of ^13^C-labeled urea and to determine useful absorbance wavelengths for replacing the FTIR spectrometer with appropriate ICLED, ICL, and/or QCL light sources (refs (28−30)), leading to possibly compact sensing systems operated, e.g., as cell phone attachments. Thereby, application scenarios beyond clinical practice may be envisaged, leading to personalized medical care devices.

While this study demonstrated the analytical capabilities of the iHWG-based system using synthetic gas mixtures, future studies will focus on validating the technology with clinical samples to further assess its performance in real-world diagnostic scenarios. However, advancing toward clinical applications entails addressing challenges, such as the miniaturization of optical components, the development of more advanced spectral analysis models, and clinical validation with real patients. Additionally, achieving a balance between cost and performance will be critical to ensuring accessibility and adoption in resource-limited healthcare settings. Despite these challenges, prior studies conducted by our group in animal models have validated the viability of similar concepts, reinforcing the potential of this technology for real-world implementation. Overcoming these barriers will facilitate the transition to compact, portable devices that enable personalized and noninvasive diagnostics of *H. pylori* infections in personal healthcare scenarios.

## Supporting Information Available

Calibration curves of ^13^CO_2_ and ^12^CO_2_ at different iHWG lengths (Figures S1 and S2), FTIR spectra for each analyte (Figures S3 and S4), a table of figures of merit for different waveguides (Table S1) and a comparison table of different methods of detection of *H. pylori* (Table S2) (PDF)

## Abbreviations

MIR: mid-infrared spectroscopy
FTIR: Fourier-transform infrared
IR: infrared
iHWG: substrate-integrated hollow waveguide
*H. pylori*: *Helicobacter pylori*
LOD: limit of detection
LOQ: limit of quantification
MCT: mercury cadmium telluride
OAPM: off-axis parabolic mirror
QCL: quantum cascade laser
ICL: interband cascade laser
ICLED: interband cascade laser light-emitting diode
