# Supplementary material for: Helicobacter pylori Breath Test via Mid-Infrared Sensor Technology
Source: ACS Sens. 2025 Feb 8;10(2):1005–10. doi: 10.1021/acssensors.4c02785 (PMC11877511; doi:10.1021/acssensors.4c02785)
Supplement: Supplementary file 1 — se4c02785_si_001.pdf [file se4c02785_si_001.pdf]

Supporting information

Helicobacter Pylori Breath Test via Mid-Infrared Sensor Technology

Gabriela Flores Rangel[a]*, Lorena Diaz de León Martinez[a], Boris Mizaikoff[a,b]

[a]Institute of Analytical and Bioanalytical Chemistry, Ulm University, Albert-Einstein-Allee 11, 89081 Ulm, Germany
[b]Hahn-Schickard, Sedanstrasse 14, 89077 Ulm, Germany

*Email: gabriela.flores-rangel@uni-ulm.de

Calibration curves for $^{13}CO_2$ using integrated hollow waveguides (iHWGs) of different lengths.

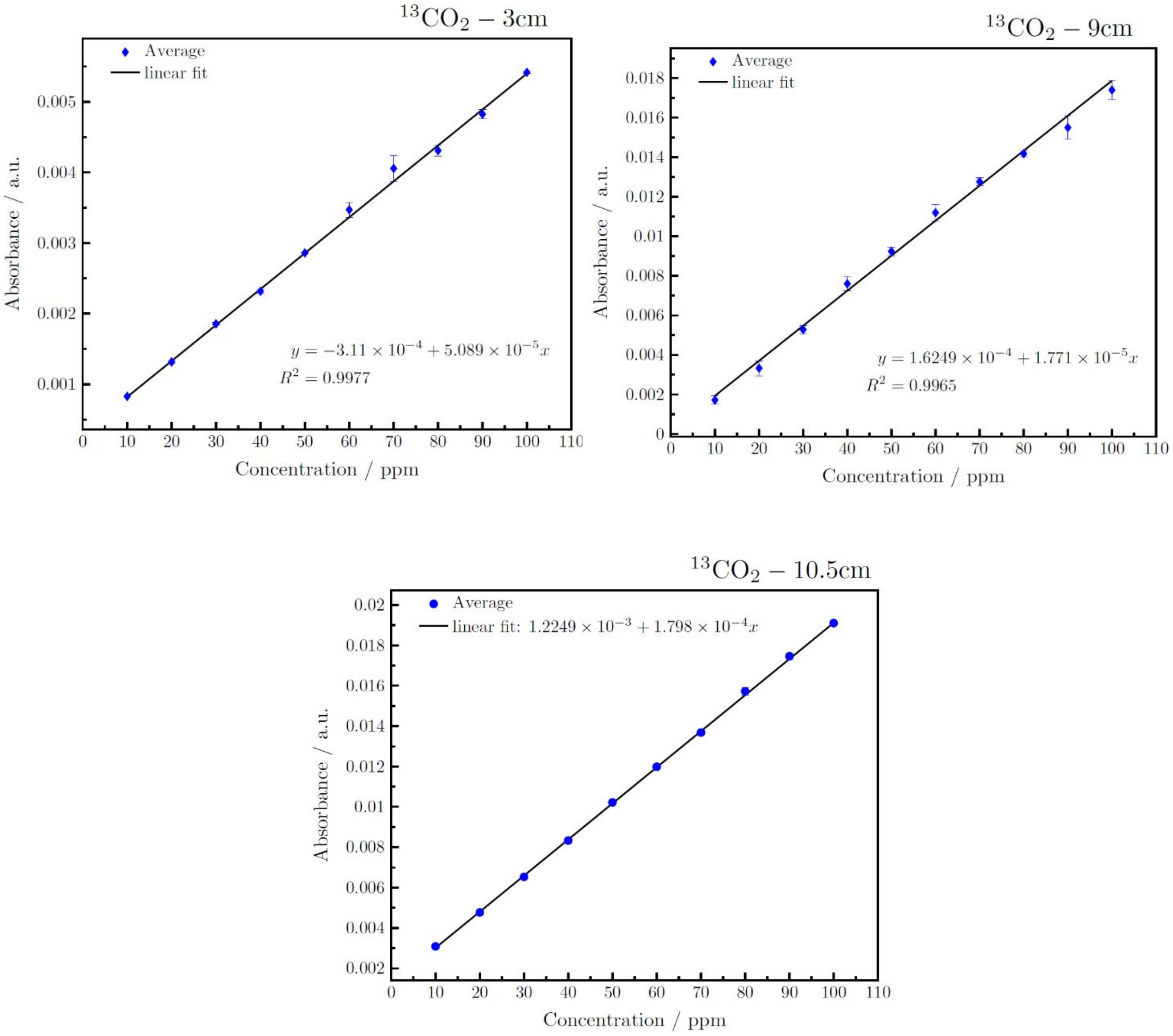

Fig S1. Calibration curves for $^{13}CO_2$ using iHWGs of 3 cm, 9 cm, and 10.5 cm in length. Measurements were conducted for concentrations ranging from 10 to 100 ppm, with linear regressions fitted for each case. The R^2 values for the linear fits are reported for each waveguide length

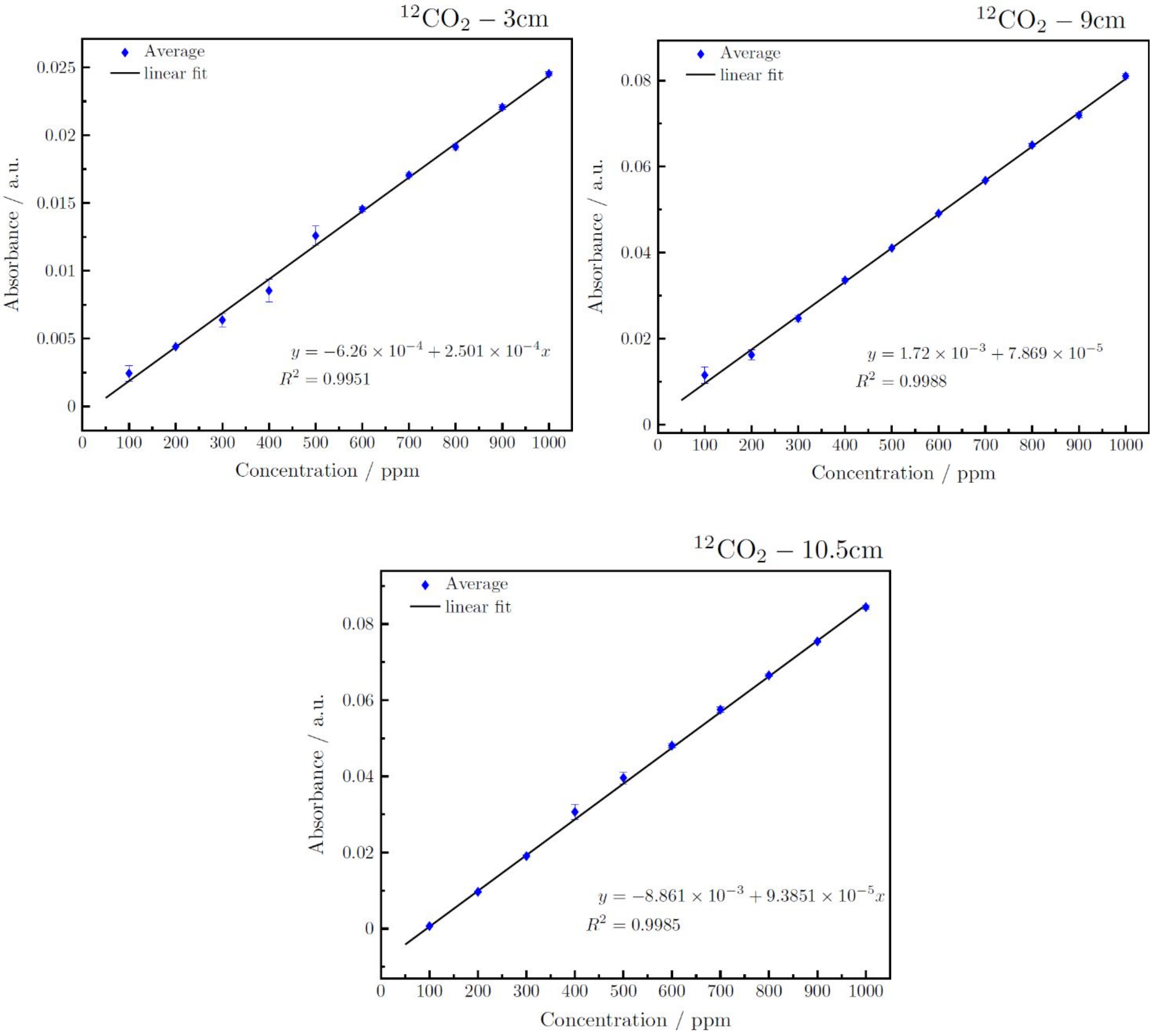

Fig S2. Calibration curves for $^{12}CO_2$ using integrated hollow waveguides (iHWGs) of 3 cm, 9 cm, and 10.5 cm in length. Measurements were conducted for concentrations ranging from 100 to 1000 ppm. The linear regressions show R^2 values of 0.9951 for 3 cm, 0.9988 for 9 cm, and 0.9985 for 10.5 cm waveguides, respectively.

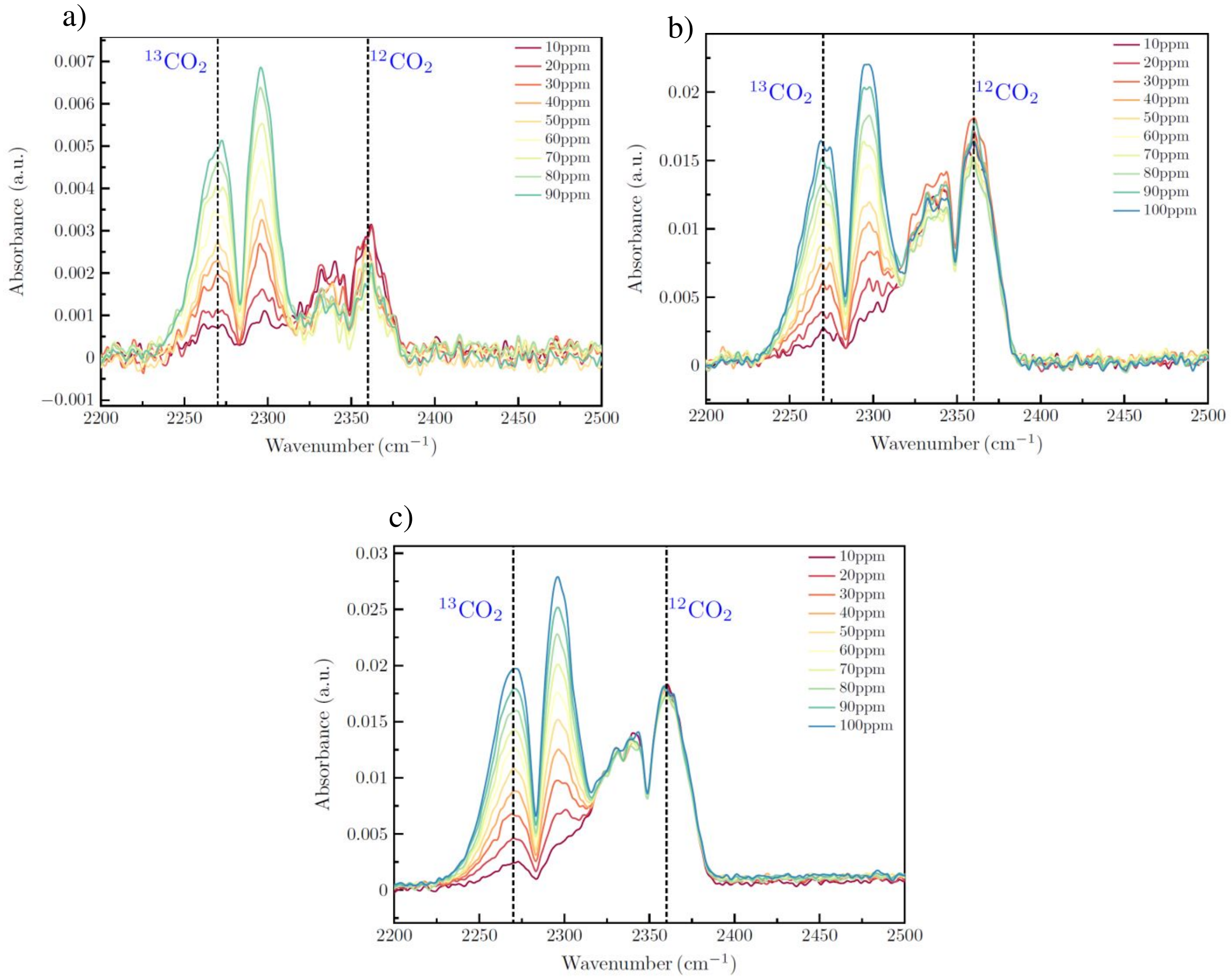

Fig S3. FTIR absorbance spectra of $^{13}CO_2$ and $^{12}CO_2$, with a fixed concentration of 200 ppm for $^{12}CO_2$ and varying concentrations of $^{13}CO_2$ (10–100 ppm). (a) Spectra obtained using a 3 cm iHWG, (b) spectra using a 9 cm iHWG, and (c) spectra using a 10.5 cm iHWG.

Table S1. Limits of detection (LOD), limits of quantification (LOQ), and linearity for $^{13}CO_2$ and $^{12}CO_2$ using different iHWG volumes across various concentration ranges.

Gas	iHWG-Volume	LOD (ppm)	LOQ (ppm)	Linearity (r^2)	Slope	Intercept
**$^{13}CO_2$ 10-100ppm**	0,48 cm^3	4.84	16.15	0.997	5.09E-05	3.25E-04
	1,31 cm^3	1.44	4.82	0.999	1.79E-04	1.19E-03
	1,44 cm^3	6.36	21.21	0.995	1.74E-04	2.11E-04
**$^{13}CO_2$ 100-1000ppm**	0,48 cm^3	37.85	126.16	0.999	4.66E-05	1.64E-03
	1,31 cm^3	90.1	300.33	0,995	1.18E-04	1.25E-02
	1,44 cm^3	79.86	266.21	0,996	1.05E-04	1.10E-0
**$^{12}CO_2$ 100-1000ppm**	0,48 cm^3	58.87	196.26	0,998	2.50E-05	-6.25E-04
	1,31 cm^3	34.48	114.96	0,999	9.33E-05	-8.19E-03
	1,44 cm^3	27.52	91.73	0,999	7.84E-05	1.92E-03

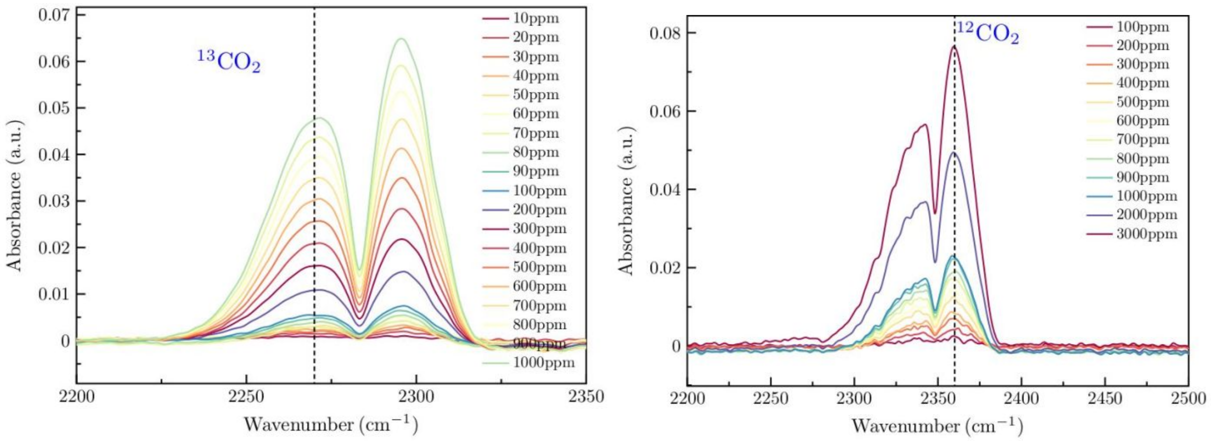

Fig S4. Absorbance spectra of $^{13}CO_2$ (10–1000 ppm) and of $^{13}CO_2$ and $^{12}CO_2$ (100–3000 ppm) using 3cm iHWG.

Table S2. Comparison of the performance of various methods for *Helicobacter pylori* detection, including the iHWG-based FTIR system from this study. Metrics such as sensitivity, specificity, limit of detection (LOD), and limit of quantification (LOQ) are summarized from relevant literature.

METHOD	SENSITIVITY	SPECIFICITY	LOD (PPM)	LOQ (PPM)	REFERENCE
**13C-UBT (IRMS)**	97%	100%	-	-	Charach et al. [1]
**13C-UBT**	98.10%	95.10%	-	-	Cardos et al. [2]
**13C-UBT**	76.20%	69.20%	-	-	Cardos et al. [2]
**14C-UBT**	96%	93%	-	-	Cardos et al. [2]
**13C-UBT**	92.5%	89.9%	-	-	K. Jambi [3]
**14C-UBT**	95.4%	97.5%	-	-	Yue-Hua et al. [4]
**13C-UBT**	97%	95%	-	-	Abd Rahim et al. [5]
**FTIR**	-	-	~1.57-4.84	~5.23-6.03	Qiu et al. [6]
**MCS**	100%	97.9%	-	-	Richter et al. [7]
**13C-UBT**	95.7%	97.9%	-	-	Motta et al. [8]
**GAS SENSOR**	-	-	200 ppb	-	Yu et al. [9]
**FTIR-IHWG (3 CM)**	-	-	4.84 (13CO2), 58.84 (12CO2)	-	This study
**FTIR-IHWG (9 CM)**	-	-	1.57 (13CO2), 19.11 (12CO2)	5.23 (13CO2), 63.70 (12CO2)	This study
**FTIR-IHWG (10 CM)**	-	-	1.81 (13CO2), 22.03 (12CO2)	6.03 (13CO2), 73.43 (12CO2)	This study

References

(1) Charach, L.; Perets, T. T.; Gingold-Belfer, R.; Huta, Y.; Ashorov, O.; Levi, Z.; Dickman, R.; Boltin, D. Comparison of Four Tests for the Diagnosis of Helicobacter Pylori Infection. *Healthcare* **2024**, *12* (15), 1479. https://doi.org/10.3390/healthcare12151479.

(2) Cardos, A. I.; Maghiar, A.; Zaha, D. C.; Pop, O.; Fritea, L.; Miere (Groza), F.; Cavalu, S. Evolution of Diagnostic Methods for Helicobacter Pylori Infections: From Traditional Tests to High Technology, Advanced Sensitivity and Discrimination Tools. *Diagnostics* **2022**, *12* (2), 508. https://doi.org/10.3390/diagnostics12020508.

(3) Jambi, L. K. Systematic Review and Meta-Analysis on the Sensitivity and Specificity of 13C/14C-Urea Breath Tests in the Diagnosis of Helicobacter Pylori Infection. *Diagnostics* **2022**, *12* (10), 2428. https://doi.org/10.3390/diagnostics12102428.

(4) Han, Y.-H.; Zhang, W.; Wang, Y.-T.; Xiong, Z.-J.; Du, Q.; Xie, Y.; Lu, H. Performance Evaluation of a Novel 14C-Urea Breath Test (Solid Scintillation) for the Diagnosis of Helicobacter Pylori Infection. *Medicine (Baltimore)* **2023**, *102* (9), e33107. https://doi.org/10.1097/MD.0000000000033107.

(5) Abd Rahim, M. A.; Johani, F. H.; Shah, S. A.; Hassan, M. R.; Abdul Manaf, M. R. ^{13}C-Urea Breath Test Accuracy for *Helicobacter Pylori* Infection in the Asian Population: A Meta-Analysis. *Ann. Glob. Health* **2019**, *85* (1), 110. https://doi.org/10.5334/aogh.2570.

(6) Qiu, M.; Liao, F.; Tan, Y.; Zhang, J.; Zheng, C.; Wang, H.; Zhuang, H.; Xiong, W.; Xie, Q.; Dong, W. Application of Fourier Transform Infrared Spectroscopy to Exhaled Breath Analysis for Detecting Helicobacter Pylori Infection. *Sci. Rep.* **2024**, *14* (1), 31542. https://doi.org/10.1038/s41598-024-83360-0.

(7) Richter, V.; Gonzalez, J. O.; Hazan, S.; Gottlieb, G.; Friedenberg, K.; Gatof, D.; Ganeshappa, R.; Delgado, J.-S.; Abramowitz, D.; Hardi, R.; Coates, A.; Haq, M.; Mehta, N.; Jones, B. A.; Moss, S. F.; Shirin, H. The Validity of Breath Collection Bags Method in Detecting *Helicobacter Pylori* Using the Novel *BreathID® Hp Lab System* : A Multicenter Clinical Study in 257 Subjects. *Ther. Adv. Gastrointest. Endosc.* **2019**, *12*, 2631774519843401. https://doi.org/10.1177/2631774519843401.

(8) Motta, O.; De Caro, F.; Quarto, F.; Proto, A. New FTIR Methodology for the Evaluation of 13C/12C Isotope Ratio in Helicobacter Pylori Infection Diagnosis. *J. Infect.* **2009**, *59* (2), 90–94. https://doi.org/10.1016/j.jinf.2009.06.006.

(9) Wu, H.; Li, D.; Liu, J.; Gong, X.; Wang, T.; Zhao, L.; Wang, T.; Yan, X.; Liu, F.; Sun, P.; Lu, G. Portable and Hand-Held Ammonia Gas Sensor Enables Noninvasive Prediagnosis of *Helicobacter Pylori* Infection. *ACS Sens.* **2024**, *9* (10), 5384–5393. https://doi.org/10.1021/acssensors.4c01609.
